# Positive Classification Advantage of Categorizing Emotional Faces in Patients With Major Depressive Disorder

**DOI:** 10.3389/fpsyg.2022.734405

**Published:** 2022-07-01

**Authors:** Lun Zhao, Xiaoyu Wang, Gang Sun

**Affiliations:** ^1^School of Education Science, Liaocheng University, Liaocheng, China; ^2^The Department of Medical Imaging, The 960^th^ Hospital of Joint Logistics Support Force of PLA, Jinan, China

**Keywords:** MDD, positive classification advantage, facial expression, configural processing, face inversion

## Abstract

This study investigated whether patients with MDD (major depressive disorder) have deficits in emotional face classification as well as the perceptual mechanism. We found that, compared with the control group, MDD patients exhibited slower speed and lower accuracy in emotional face classification. In normal controls, happy faces were classified faster than sad faces, i.e., positive classification advantage (PCA), which disappeared under the inverted condition. MDD patients showed PCA similar to the control group, although the inversion effects of happy and sad faces were more evident. These data suggest that the dysfunction of categorizing emotional faces in MDD patients could be due to general impairment in decoding facial expressions, reflecting the more common perceptual motion defects in face expression classification.

## Introduction

Patients with major depressive disorders (MDD) have defects in recognizing facial expressions. This kind of facial expression recognition difficulty already exists in children with depression, and even if the individual recovers from the onset of depression, this kind of difficulty still exists (Lee et al., 2016). Importantly, these deficits may lead to a decline in social cognitive function and may be associated with subsequent recurrence of depression. According to the cognitive theory of depression, in the process of the onset, maintenance, and recurrence of MDD, there is cognitive preference in processing emotional stimuli, which plays an important role in the development and persistence of depression (e.g., Joormann and Gotlib, 2006; Venn et al., 2006).

Previous studies have shown that MDD patients have specific impairment of positive expression cognition. For example, Surguladze et al. (2004) found that, compared with the control group, MDD patients were less likely to label 50% of distorted happy faces with happiness, indicating that MDD patients had difficulty in recognizing mild happy expressions. Gotlib et al. (2004) showed the participants facial images that slowly changed from a neutral expression to a fully emotional expression, and found that, compared with the control group, MDD patients need higher intensity to correctly recognize happy faces. Using the forced choice task, in which participants were asked to choose which of the two faces showed stronger emotion, Joormann and Gotlib (2008) showed that MDD patients could not judge subtle happy expressions as stronger than neutral and negative expressions and that when happy expressions and negative expressions (such as anger, fear, or sadness) appeared at the same time, MDD patients were less likely to choose happy faces as stronger emotional expression, indicating that the difficulty of positive emotion detection in MDD patients may lead to their lack of strong feeling and reduce their approach behavior.

Different from the attention, memory, and recognition of emotional faces, the simple classification of facial expressions is mainly based on the experience and top-down processing of common feature attribute template. It is found that, in healthy people, positive facial expressions (i.e., happy expression) are categorized faster than negative facial expressions (such as sadness, anger, etc.), which is referred to as positive classification advantage (PCA) (Leppänen and Hietanen, 2004). This classification advantage is not related to the early stage of face processing reflected by the N170 of ERP components, but related to late classification processing reflected by the P3 component (Liu et al., 2013; Song et al., 2017; Yan et al., 2018). Converging evidence has found that MDD patients have cognitive dysfunction in facial expression judgment and recognition. For example, there was evidence that MDD patients completed emotion classification tasks with much slower speed and could not accurately recognize the subtle changes of other people's facial expressions in the social environment, which may be the basis of the impairment of interpersonal function in depression (Leppänen et al., 2004). Surguladze et al. (2005) asked participants to identify sad, happy, and neutral facial expressions with different presentation times (100 and 2,000 ms) and found that, compared with the control group, MDD patients only showed slight differences in the accuracy of identification, but showed obvious deficiencies in the identification of mild happy expressions. On the contrary, there was evidence that the classification accuracy of happy expressions in MDD patients was better than all other expressions such as angry, sad, neutral, and surprised faces (Conte et al., 2018). To date, the research results on the process of facial expressions in MDD patients, especially processing sad faces, are actually inconsistent. However, these studies did not pay attention to the face classification by expression *per se*, that is, they did not systematically observe whether there is PCA phenomenon as well as its perceptual mechanism in MDD patients. In fact, previous studies have shown that the expression processing disorder is affected by task goals in MDD patients. For example, Leppänen and Hietanen, 2004 found that, in the expression matching task (identifying whether the third face matches the first face or the second face), the MDD patients did not show any difference from the control group, but in the emotion recognition task, they had a longer reaction time to the sad emotion.

In addition, the main source for PCA is based on global/configural processing, that is, when the spatial arrangement of the face structure is disturbed (such as face inversion), the PCA weakens or disappears (Song et al., 2017). Recently, de Fockert and Cooper (2014) used the Navon task, a standard task investigating local and holistic processing, to study the relationship between depression and visual processing. They found that the participants with low depression showed global processing bias, i.e., the response to global information was significantly faster, but the participants with high depression did not observe the difference, suggesting that depression was related to the decrease of global processing advantage. The present study would directly explore the PCA in MDD patients as well as the influence of face inversion on expression classification. Specifically, we would explore whether patients with depression have decreased or enhanced PCA. We would also determine the perceptual mechanism of PCA in depression, that is, local processing or global/configural processing.

## Methods

### Participants

In this study, we recruited 32 MDD patients (17 females; 22–45 years, mean 33.5 ± 9.6 years) from the outpatient department in the 960th Hospital, China. Each patient was admitted for a psychiatry evaluation for the first time in their lives, and had never used any antidepressants, anxiolytic drugs, or antipsychotic drugs according to the interview and hospital records. Thirty-four age-matched healthy control participants were recruited by social recruitment (18 females; 20–45 years, mean 32.9 ± 9.9 years; *p* = 0.25; Table 1)^1^. According to DSM-IV (Diagnostic and Statistical Manual of Mental Disorders, Fourth Edition), each patient was diagnosed with MDD. Patients with a history of mental or neurological disorders were excluded. The exclusion criteria also included head trauma, learning disabilities, bipolar disorder, and alcohol or drug abuse.

**Table 1:** Demographic and clinical characteristics of major depressive disorder (MDD) and healthy controls (HC).

	**MDD (*n* = 32)**	**HC (*n* = 34)**	***P***
Gender (M/F)	15/17	16/18	0.88
Age (y)	33.5	32.9	0.25
Education (y)	13.9	13.5	0.89
Handedness (Left/Right)	32/0	34/0	
HRSD-17	23.16	2.49	0.000
HAMA	20.38	2.57	0.000
MMSE (Mini-mental State Examination)	29.6	29.2	0.86

Hamilton Depression Rating Scale 17 item scores (HRSD-17) were used to assess the severity of depression (23.16 ± 3.29 and 2.49 ± 1.08 in patients and controls, respectively; *p* = 0.000) and 14 item Hamilton Anxiety Scale (HAMA) scores showed the severity of comorbid anxiety (20.38 ± 3.18 and 2.57 ± 1.05 in patients and controls, respectively; *p* = 0.000). According to the score of MMSE (Mini-mental State Examination), there was no obvious cognitive impairment in the depression group (29.6 ± 3.56 and 29.2 ± 3.32 in patients and controls, respectively; *p* = 0.86). During this study, no patients took antidepressants, mood stabilizers, antipsychotics, anxiolytics, and hypnotics.

The healthy control group did not have any history of major mental or physical diseases, and did not take any drugs that affect the nervous system. All participants were given a certain reward and provided their informed consent before the experiment, which was approved by the Ethics Committee of the 960th Hospital, China.

### Stimuli and Procedure

**Figure 1:**
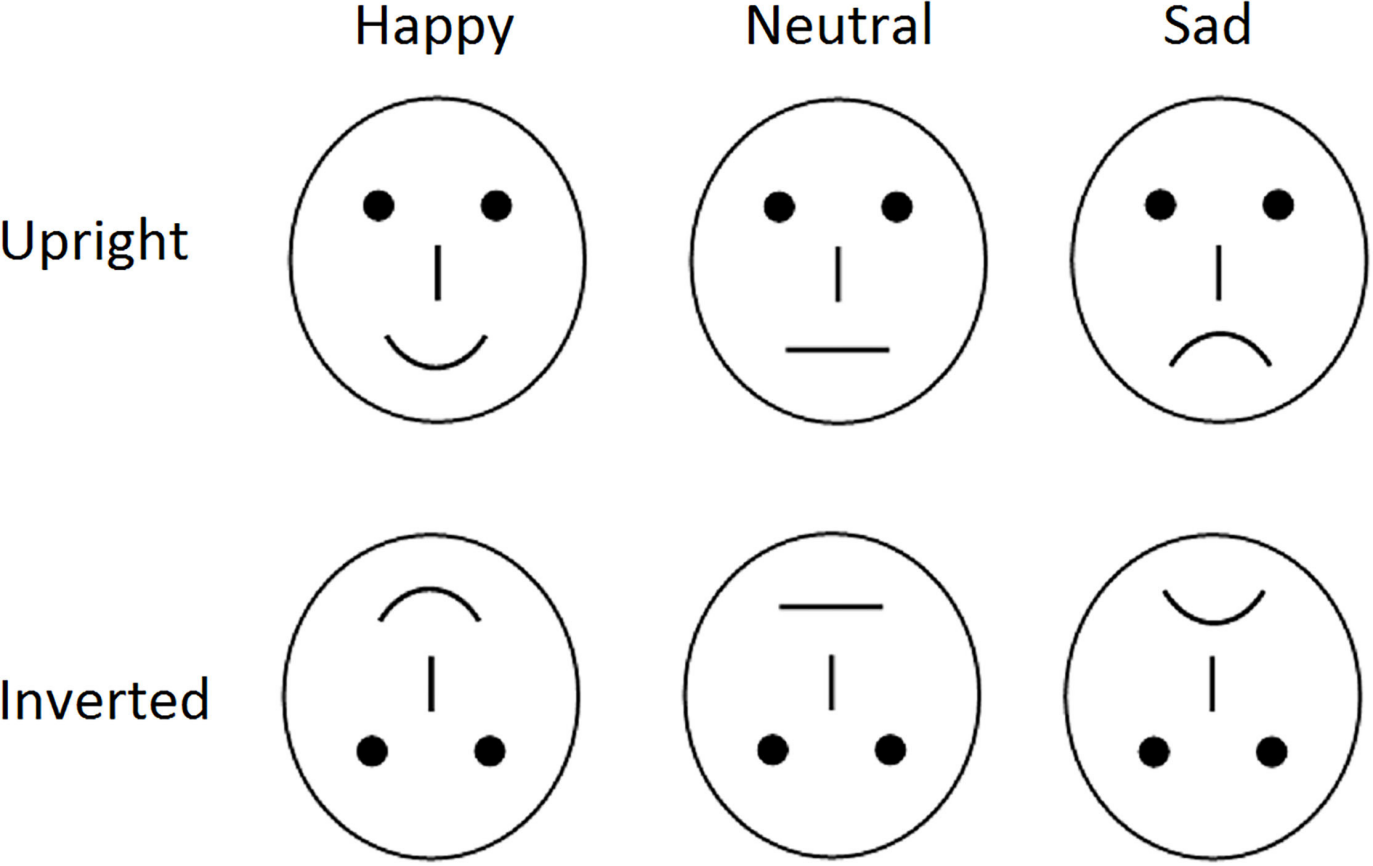
Schematic stimuli used in the present study.

In order to avoid the low-level processing of facial features as well as boredom by the excessive repetition of one single model, using Adobe Photoshop CS6 we constructed each emotional category to consist of 40 different schematic face models by manipulating the distance among facial features and by manipulating the shape of the facial features. For example, the distortion of second order relations in each of the face stimuli were made by reducing the distance between the eyes by 10–20%, lowering the eyes and the eyebrows level relative to the tip of the nose by 10–20%, or reducing the distance between the root of the nose and the mouth by 10–20%. Three types of emotional faces, i.e., happy, sad, and neutral facial expressions, were included (Figure 1). Each type of emotional faces included 40 different pictures which were randomly presented in upright and inverted conditions, with a total of 240 face pictures (120 upright faces, 40 for each type of facial expression; 120 inverted faces, 40 for each type of facial expression). All stimuli were displayed in the center of the video monitor; the viewing distance was 100 cm, with the vision angle of about 7.27° × 6.06°.

The 240 stimuli were divided into two blocks (120 faces for each) and presented randomly. The participants were asked to press the corresponding buttons on the keyboard, i.e., “X” by left index finger, “N” and “M” by right index finger, representing happy, sad, or neutral, respectively, as quickly and correctly as possible to classify the expressions of each face. The reaction buttons (e.g., “X” = happy, “N” = sad, “M” = neutral) were cross balanced in the participants. Each stimulus lasted for 300 ms and, after the response, the next trial was presented after a random interval of 600–800 ms. There was a short break between two blocks. Before the formal experiment, there was an exercise sequence with 36 stimuli (six faces in each stimulus category), which were not presented in the following experiment.

### Data Analysis

Response time (RT) and response accuracy were analyzed by a three-way ANOVA, with Expression (happy, sad, neutral) and Orientation (upright, inverted) as within-subject factors and Group (controls, patients) as the between-subject factor. Degrees of freedom were adjusted using the Greenhouse–Geisser epsilon correction factor for all ANOVAs and pairwise comparisons used Bonferroni corrections for multiple comparisons.

## Results

For each subject, the trials with RTs more than ±2 *SD* were excluded. Table 2 showed the classification performance of emotional faces in controls and MDD patients, respectively. The results of statistical analysis were as follows:

**Table 2:** The accuracy rate (%) and reaction times (RTs) of classification of emotional faces in healthy controls (HC) and MDD patients, respectively.

**Condition**	**Expression**	**Accuracy (% ±SD)**		**RTs (ms ±SD)**	
		**HC**	**MDD**	**HC**	**MDD**
Upright	Neural	98.9 ± 1.2	93.5 ± 4.6	635 ± 71	756 ± 89
	Happy	95.6 ± 2.4	90.8 ± 5.5	681 ± 89	855 ± 131
	Sad	96.2 ± 2.8	91.1± 4.8	768 ± 96	935 ± 188
Inverted	Neural	97.9 ± 2.2	89.8 ± 5.2	640 ± 77	755 ± 84
	Happy	91.6 ± 4.2	83.8 ± 6.7	852 ± 121	1,138 ± 211
	Sad	91.5 ± 4.6	84.2 ± 6.2	846 ± 108	1,146 ± 256

For the analysis of accuracy, the main effect of Group was significant [*F*_(1, 64)_ = 4.02, *p* = 0.049], Partial η^2^ = 0.198, showing lower accuracy in MDD (88.9%) than controls (95.4%). The face inversion significantly disturbed facial expression classification, [*F*_(1, 64)_ = 5.13, *p* = 0.027], Partial η^2^ = 0.118, showing decreased accuracy in inverted (89.8%) over upright (94.4%) conditions. The main effect of Expression was also significant, [*F*_(2, 128)_ = 3.58, *p* = 0.031], Partial η^2^ = 0.139, revealing that neutral face classification was categorized more accurately (95.0%) than happy (90.5%, *p* = 0.026) and sad faces (90.8%, *p* = 0.035), but there was no significant difference between the latter two conditions (*p* = 0.87). The two-way interaction of Orientation ^*^ Expression was significant, [*F*_(2, 128)_ = 3.98, *p* = 0.021], Partial η^2^ = 0.159 and further analysis showed that face inversion had a slight effect on the accuracy of classifying neutral faces (96.2 and 93.9% for upright and inverted conditions, respectively; *p* = 0.68), but significantly reduced the classification accuracy of happy and sad faces (93.2 and 87.7% for upright and inverted happy faces, respectively, *p* = 0.028; 93.7 and 87.9% for upright and inverted sad faces, respectively, *p* = 0.032). Other effects were not significant.

Similar to the analysis of accuracy, the ANOVA of RTs also showed a significant main effect of Group, [*F*_(1, 64)_ = 9.89, *p* = 0.0025], Partial η^2^ = 0.156, showing slower reaction speed in MDD (931 ms) than controls (737 ms). The face inversion significantly decreased the speed of categorizing facial expression, [*F*_(1, 64)_ = 8.25, *p* = 0.006], Partial η^2^ = 0.153 (772 and 896 ms for upright and inverted conditions, respectively). The main effect of Expression was also significant, [*F*_(2, 128)_ = 4.05, *p* = 0.020], Partial η^2^ = 0.159, revealing that neutral face classification was categorized more quickly (697 ms) than happy (882 ms, *p* = 0.038) and sad faces (924 ms, *p* = 0.028), but the latter two conditions did not reach significant difference, although the classification speed of a happy face was faster than a sad face (*p* = 0.079). The interaction between Orientation and Expression was significant, [*F*_(2, 128)_ = 6.08, *p* = 0.003], partial η^2^ = 0.098. Further analysis revealed that the speed of neutral face classification was not modulated by face inversion (696 and 698 ms for upright and inverted conditions, respectively; *p* = 0.89), but face inversion significantly delayed the classification speed of happy (768 and 995 ms for upright and inverted faces, *p* = 0.016) and sad faces (852 and 996 ms for upright and inverted faces, *p* = 0.028). Although there was no significant difference of the inversion effect between the happy (inversion effect: 227 ms) and sad faces (134 ms, *p* = 0.085), the PCA was significant in upright condition (sad minus happy: 84 ms, *p* = 0.035; controls: 87 ms, *p* = 0.036; MDD: 80 ms, *p* = 0.039), but absent in inverted condition (3 ms, *p* = 0.86; controls: −6 ms, *p* = 0.58; MDD: 8 ms, *p* = 0.46). Although the Expression ^*^ Group interaction was not significant [F_(2, 128)_ = 0.93], interestingly, we did find a significant three-way interaction of Expression ^*^ Orientation ^*^ Group, [*F*_(2, 128)_ = 6.26, *p* = 0.0025], Partial η^2^ = 0.109. Further analysis of this three-way interaction effect showed that, although the classification of neutral expression in MDD patients was significantly slower than that in the control group, there was no Orientation ^*^ Group interaction [*F*_(1, 64)_ = 0.68], that is, neutral faces did not show inversion effect in both two groups [*F*_(1, 31)_ = 0.58 and F_(1, 33)_ = 0.78 for MDD patients and controls, respectively]. The main effects of Orientation for both happy and sad faces were qualified by Group, that is, the inverted effect was larger in patients (283 and 211 ms for happy and sad faces, respectively) than in controls [171 and 98 ms for happy (*p* = 0.028) and sad (*p* = 0.036) faces, respectively]. Other effects were not significant.

## Discussion

In the present study, we directly explored the phenomenon of positive classification advantage (PCA) related to facial expression classification as well as the perceptual mechanism reflected by face inversion in MDD patients (see also, Ridout et al., 2003; Tong et al., 2020). The results showed that the classification of happy faces was faster than that of sad faces in normal control group, i.e., PCA, and the PCA disappeared under the inverted condition. Compared with the control group, the overall classification of emotional faces was slower and the accuracy was lower in MDD patients. However, the PCA effect was similar in MDD patients to that in the controls, although the inversion effects of happy and sad expressions were more evident than in the controls.

In line with previous studies, the present results showed that the classification of happy faces was faster than that of sad faces, i.e., PCA, and this effect disappeared when faces were inverted (Leppänen and Hietanen, 2004; Liu et al., 2013; Song et al., 2017; Xu et al., 2020). It is generally believed that the inversion of the human face will weaken its overall spatial structure and feature information, thus interfering with overall face processing. Therefore, the difference of configural computation between happy and sad faces could be one of the sources accounting for faster classification of happy faces (Leppänen and Hietanen, 2004; Song et al., 2017).

Converging evidence confirmed that the cognitive bias to other people's negative emotion in MDD patients was increased, i.e., negative cognitive bias (Bouhuys et al., 1999; Yoon et al., 2009; Beevers et al., 2015; Scibelli et al., 2016). Inconsistent with the above negative cognitive bias, although the expression classification of faces was slower and the accuracy was lower in MDD patients, i.e., the dysfunction of categorizing facial expressions, we did not find the interaction effect of Group ^*^ Expression, suggesting that there was a similar reaction pattern of expression classification between MDD and healthy controls. Similar to the present findings, Karparova et al. (2005) did not find attentional biases for emotional faces in MDD patients, suggesting that MDD patients have general impairment in decoding facial expressions, regardless of the type of expressions. Therefore, the increase of response time and error rate in MDD patients may reflect the more common perceptual defects in the specific processing of facial expressions.

Interestingly, although there was similar PCA between MDD and controls, the inversion effect of emotional faces was more evident in MDD patients than in controls, indicating that MDD patients do process the global/configural information of facial expressions, and may be more dependent on the global/configural processing in facial expression classification than normal controls. However, contrary to the present results, previous studies found that MDD patients tend to pay attention to details and individual information, rather than the global information, which is correlated to depression symptoms. Recently, using the Navon task, a standard task for local and global processing, de Fockert and Cooper (2014) found that the participants with low depression showed faster responses to global processing, i.e., global processing bias, but the participants with high depression did not observe this effect, suggesting that depression was related to the decrease of global processing advantage. In addition, Basso et al. (1996) found that the global processing was positively correlated with the individual trait of wellbeing and negatively correlated with the individual trait of depression. However, the present study also found that, compared with controls, MDD patients showed a similar inversion effect on the classification of neutral facial expressions, indicating that the classification and processing of neutral faces has a relatively complete perceptual processing mechanism in MDD patients. To our knowledge, this is the first study on the cognitive mechanism of expression classification processing in MDD patients and the results of more obvious inversion effects are not consistent with previous studies on the disorder of global processing priority. Although the inconsistency of tasks in previous studies may be one of the reasons, the basic mechanism of this phenomenon needs further study.

In conclusion, this study investigated emotional face classification as well as its perceptual mechanism in MDD patients. We found that, compared with the control group, the classification speed of emotional faces in MDD patients was slower and the accuracy was lower. In the control group, happy faces were classified faster than sad faces, i.e., PCA, which disappeared under the inverted condition. MDD patients exhibited similar PCA to the control group, although the inversion effects of happy and sad expressions were more evident than in the control group.

## Data Availability Statement

The raw data supporting the conclusions of this article will be made available by the authors, without undue reservation.

## Ethics Statement

The studies involving human participants were reviewed and approved by Ethics Committee of the 960th Hospital, China. The patients/participants provided their written informed consent to participate in this study.

## Author Contributions

LZ finished data collection and the draft. XW finished data collection. GS finished the design and revision. All authors contributed to the article and approved the submitted version.

## Funding

This work was supported by the Basic Research Key Program, Defence Advanced Research Projects of PLA (2019-JCQ-ZNM-02).

## Conflict of Interest

The authors declare that the research was conducted in the absence of any commercial or financial relationships that could be construed as a potential conflict of interest.

## Publisher's Note

All claims expressed in this article are solely those of the authors and do not necessarily represent those of their affiliated organizations, or those of the publisher, the editors and the reviewers. Any product that may be evaluated in this article, or claim that may be made by its manufacturer, is not guaranteed or endorsed by the publisher.

## Footnotes

^1^In the present study, we used G PowerWin 3.1 to sample size calculation of data (F tests) with effect size d of 0.4, α err prob of 0.05 and power (1-β err prob) of 0.8 and showed that the current sample sizes of 32 and 34 for MDD and control groups, respectively, were acceptable for this study (Faul et al., 2009).

## References

[B1] Basso M. R. Schefft B. K. Ris M. D. Dember W. N. (1996). Mood and global-local visual processing. J. Int. Neuropsychol. Soc. 2, 249–255. 10.1017/S1355617700001193 9375191

[B2] Beevers C. G. Clasen P. C. Enock P. M. Schnyer D. M. (2015). Attention bias modification for major depressive disorder: effects on attention bias, resting state connectivity, and symptom change. J. Abnorm. Psychol. 124, 463–475. 10.1037/abn0000049 25894440 PMC4573770

[B3] Bouhuys A. L. Geerts E. Gordijn M. C. (1999). Depressed patients' perceptions of facial emotions in depressed and remitted states are associated with relapse: a longitudinal study. J. Nerv. Ment. Dis. 187, 595–602. 10.1097/00005053-199910000-00002 10535652

[B4] Conte S. Brenna V. Ricciardelli P. Turati C. (2018). The nature and emotional valence of a prime influences the processing of emotional faces in adults and children. Int. J. Behav. Dev. 42, 554–562. 10.1177/0165025418761815

[B5] de Fockert J. W. Cooper A. (2014). Higher levels of depression are associated with reduced global bias in visual processing. Cogn. Emot. 28, 541–549. 10.1080/02699931.2013.839939 24067089

[B6] Faul F. Erdfelder E. Buchner A.. (2009). Statistical power analyses using G^*^ Power 3. 1: tests for correlation and regression analyses. Behav. Res. Methods 41, 1149–1160. 10.3758/BRM.41.4.1149 19897823

[B7] Gotlib I. H. Krasnoperova E. Yue D. N. Joormann J. (2004). Attentional biases for negative interpersonal stimuli in clinical depression. J. Abnorm. Psychol. 113, 127–135. 10.1037/0021-843X.113.1.121 14992665

[B8] Joormann J. Gotlib I. H. (2006). Is this happiness I see? Biases in the identification of emotional facial expressions in depression and social phobia. J. Abnorm. Psychol. 115, 705–714. 10.1037/0021-843X.115.4.705 17100528

[B9] Joormann J. Gotlib I. H. (2008). Updating the contents of working memory in depression: interference from irrelevant negative material. J. Abnorm. Psychol. 117, 182–192. 10.1037/0021-843X.117.1.182 18266496

[B10] Karparova S. P. Kersting A. Suslow T. (2005). Disengagement of attention from facial emotion in unipolar depression. Psychiatry Clin. Neurosci. 59, 723–729. 10.1111/j.1440-1819.2005.01443.x 16401250

[B11] Lee S. A. Kim C. Y. Lee S. H. (2016). Non-conscious perception of emotions in psychiatric disorders: the unsolved puzzle of psychopathology. Psychiatry Investig. 13:165. 10.4306/pi.2016.13.2.165 27081376 PMC4823191

[B12] Leppänen J. M. Hietanen J. K. (2004). Positive facial expressions are recognized faster than negative facial expressions, but why? Psychol. Res. 69, 22–29. 10.1007/s00426-003-0157-2 14648224

[B13] Leppänen J. M. Milders M. Bell J. S. Terriere E. Hietanen J. K. (2004). Depression biases the recognition of emotionally neutral faces. Psychiatry Res. 128, 123–133. 10.1016/j.psychres.2004.05.020 15488955

[B14] Liu X. F. Liao Y. Zhou L. Sun G. Li M. Zhao L. (2013). Mapping the time course of the positive classification advantage: an ERP study. Cogn. Affect. Behav. Neurosci. 13, 491–500. 10.3758/s13415-013-0158-6 23504806

[B15] Ridout N. Astell A. Reid I. Glen T. O'Carroll R. (2003). Memory bias for emotional facial expressions in major depression. Cogn. Emot. 17, 101–122. 10.1080/02699930302272 29715743

[B16] Scibelli F. Troncone A. Likforman-Sulem L. Vinciarelli A. Esposito A. (2016). How major depressive disorder affects the ability to decode multimodal dynamic emotional stimuli. Front. ICT 3:16. 10.3389/fict.2016.00016

[B17] Song J. Liu M. Yao S. Yan Y. Ding H. Yan T.. (2017). Classification of emotional expressions is affected by inversion: behavioral and electrophysiological evidence. Front. Behav. Neurosci. 11:21. 10.3389/fnbeh.2017.00021 28232793 PMC5298963

[B18] Surguladze S. Brammer M. J. Keedwell P. Giampietro V. Young A. W. Travis M. J.. (2005). A differential pattern of neural response toward sad versus happy facial expressions in major depressive disorder. Biol. Psychiatry 57, 201–209. 10.1016/j.biopsych.2004.10.028 15691520

[B19] Surguladze S. A. Young A. W. Senior C. Brébion G. Travis M. J. Phillips M. L. (2004). Recognition accuracy and response bias to happy and sad facial expressions in patients with major depression. Neuropsychology 18, 212–218. 10.1037/0894-4105.18.2.212 15099143

[B20] Tong Y. Zhao G. Zhao J. Xie N. Yang Y. (2020). Biases of happy faces in face classification processing of depression in Chinese patients. Neural Plast. 2020:7235734. 10.1155/2020/7235734 32879624 PMC7448107

[B21] Venn H. Watson S. Gallagher P. Young A. H. (2006). Facial expression perception: an objective outcome measure for treatment studies in mood disorders? Int. J. Neuropsychopharmacol. 9, 229–245. 10.1017/S1461145705006012 16316484

[B22] Xu S. Liu X. Zhao L. (2020). Categorization of emotional faces in insomnia disorder. Front. Neurol. 11:569. 10.3389/fneur.2020.00569 32636799 PMC7317303

[B23] Yan T. Dong X. Mu N. Liu T. Chen D. Deng L.. (2018). Positive classification advantage: tracing the time course based on brain oscillation. Front. Hum. Neurosci. 11:659. 10.3389/fnhum.2017.00659 29375353 PMC5768652

[B24] Yoon K. L. Joormann J. Gotlib I. H. (2009). Judging the intensity of facial expressions of emotion: depression-related biases in the processing of positive affect. J. Abnorm. Psychol. 118, 223–228. 10.1037/a0014658 19222328 PMC2835523

